# Cynanche Laryngea, or Acute Laryngitis

**Published:** 1838-01-31

**Authors:** N. Chapman

**Affiliations:** Professor of the Theory and Practice of Physic, in the University of Pennsylvania


THE
### MEDICAL EXAMINER.

DEVOTED TO MEDICINE, SURGERY AND THE COLLATERAL SCIENCES.

EDITED by J. B. BIDDLE, M. D. and M. CLYMER, M. D.

No. 3. PHILADELPHIA, WEDNESDAY, JANUARY 31, 1838. Vol. I.

### CYNANCHE LARYNGEA,
### OR ACUTE LARYNGITIS.

*By* N. Chapman, M. D., *Professor of the Theory and Practice of Physic, in the University of Pennsylvania.*

The first regular account of this disease is contained in a paper by Sir Matthew Baillie, in the third volume of the Transactions of the Society for the improvement of Medical and Chirurgical knowledge, for the year 1812. It has since attracted considerable attention, and not a few cases of it are reported in the periodical journals of Europe and elsewhere. The curiosity thus excited, has led to researches into the older writers, and it appears probably to have existed even from the earliest times. It is alleged to have been noticed by Hippocrates, Celsus, Galen, Cœlius, Aurelianus, Lommius, Tulpius, Riverius, Morgagni, Boerhaave, Lieutaud, and Mead. But their accounts are slight, vague and indistinct, and on the whole, the credit of the first accurate description of it, is accorded with sufficient unanimity to the communication by Baillie, to which I have alluded. It is, however, only justice to state, that a distinguished physician of our own country, the late Dr. Dick, seems to have recognized the disease, at a somewhat earlier date, having in a well written paper on Croup, published in 1808,* clearly referred to it, as the very worst form of that affection, and, as confined to the Larynx, denominates it Cynanche Laryngea. The number of cases of it observed, since medical attention has been more carefully directed to it, would indicate rather a want of pathological precision, than the unfrequency of it, or, that the two affections having many symptoms in common, it was up to the period mentioned, generally confounded with Croup. Yet it must be deemed a rare disease.

Laryngitis usually commences with chilliness or rigors, soon followed by a sense of dryness, or huskiness of the throat, inducing a frequent hawking, as it were to get rid of some extraneous cause of irritation. Expectoration is not regularly performed, the sputa being raised up in the manner described. Breathing at the same time is somewhat impeded, and, on a deep inspiration, which is required to inflate the lungs, a painful constriction is felt in the larynx, attended by a sibilous or whistling noise, more or less sonorous, resembling the rushing of air through a contracted aperture. It is a sound very different from the sharp thrilling intonation of croup, which never occurs, nor does regular cough prevail to any extent, at this or any subsequent period, as far as I have seen, though the reverse is affirmed. The late Dr. Armstrong however, concurs with me on this point, and states, that one of the most characteristic symptoms, is an inability to cough, the endeavour to do so terminating in a sort of grumbling or grunting noise in the throat. The voice however, is slightly hoarse, and, in some instances, though rarely at so early a period, so much affected as to degenerate into a thick or low whisper. By an effort to cough or spit, and still more by pressure, the pain in the larynx is much increased, especially at the pomum Adami, though on inspection, we usually discover only slight inflammation of the fauces, with scarcely any tumefaction of the tonsils, or other soft parts. Difficulty of deglutition however in some degree, is apt to be experienced. These symptoms are attended with more or less fever, which however, is seldom high, or the pulse full, or hard and corded, except when it begins in the shape of tonsilitis, which sometimes happens.

In other instances, the approach of the disease is milder, and more insidious, in the guise of ordinary catarrh, creating no anxiety. Little here is complained of except a slight soreness of the throat, with scarcely even an erubescence, or mere blush of inflammation, unattended by any very evident constitutional disturbance. This state of things will remain sometimes for several days, when, very suddenly a sense of severe spasmodic constriction of the larynx supervenes, and henceforward, the case loses all ambiguity,—developing, in rapid succession, those phenomena which I have already noticed.

Cases too, I have remarked, where the preliminary symptoms seem to be altogether those of gastric irritation. The stomach here, after some previous oppression becomes suddenly disordered, violent puking ensues, sometimes of bile, with little or no nausea, and, in one instance, of as limpid fluid as in pyrosis, attended by a constant discharge from the mouth, like that in the deepest salivation. During this period, though a slight soreness of throat exists, compared to the sensation of a scald, there is no appreciable laryngeal affection, or res-

* Barton's Med. and Physical Journal, vol. III.

piratory embarrassment in any way. But all at once, as it were, a translation seems to take place from the mucous tissue of the stomach to the larynx, leaving the one organ quiescent, and placing the other in a state of well marked laryngitis.

As another mode of attack, it remains to be mentioned, that commencing very much as tonsilitis, phlogosis of the highest degree rapidly extends to the velum pendulum palati, up the posterior nares, the Eustachian tube, and down into the pharynx, and larynx, implicating, in short, the whole of the neighbouring parts.

In some instances, however, this structure, instead of active phlogosis, presents, even at an early period, a pale and bloated aspect, owing to serous effusion into the sub-cellular tissue, giving to this variety of the disease, the title of Laryngitis Œdematosa.

Laryngitis, in its progress, whatever may be the manner of its invasion, assumes a graver character, mostly by an aggravation of the symptoms already detailed. The pulse now sinks or becomes diminutive, the skin is cold, collapsed, pallid, or mottled—there is great difficulty of swallowing, amounting sometimes to actual impossibility, and, in the effort, violent spasmodic strangling, as in hydrophobia, is excited, owing apparently to the fluid entering the larynx, from the epiglottis ceasing to act as a valve, it being swollen and erect.* The dyspnœa is excessive, caused by the obstruction of the glottis, or effusion into the Bronchia and cellular structure, accompanied by occasional convulsive exacerbations, productive of dreadful agitation. In this paroxysmal state, so extreme is the embarrassment of respiration, that the larynx and trachea may be seen to move quickly, upwards and downwards in the neck, and all the muscles, subservient to this function, are thrown into an irregular action, so that the chest heaves violently accompanied by a general distortion of features.† The eyes are wild and protruded, the mouth open, gasping for breath—the lips are pale or livid—and such, too, is occasionally the face, covered with cold, pearly drops of sweat—the tongue is thrust out, and swelled, and the voice scarcely audible. There is tossing of the arms, with every other indication of the utmost agony, which indeed is sometimes so intolerable, that we are told by Bayle, attempts at suicide have been known. These convulsive paroxysms being over, considerable mitigation of suffering, and disposition to sleep ensue, which respites however, are not of any long continuance, and finally death takes place in one of these exacerbations from suffocation, often with the full retention of the intellectual faculties.

As to the duration of the disease, the average is from two to five days, though some cases of it have terminated in a few hours.

The preceding sketch of Laryngitis has been drawn in the strongest colours, particularly as regards the advanced stages. Its attacks however, are occasionally milder, and, indeed, there is to be found, in its several presentations, every gradation of violence and variety of modification, from mere hoarseness, or impairment of voice, to the tremendous intensity of affection I have described.

It is a common opinion, that laryngitis prevails more frequently among males than females, and in adult, and even advanced, than early life. Certain it is, of the cases recorded, much the larger proportion was males, and far beyond the meridian of existence. That of the illustrious Washington was one of these, who died in his sixty-eighth year, and the very first instances to which Baillie was called, occurred in two eminent practitioners‡ nearly as old.

Conspicuous examples of it, however, in females, are not wanting, among the most so of which, is that of Josephine the late Empress of France, whose chequered and eventful life was thus closed in her forty-eighth year. My own practice has supplied me with several instances of it in females beyond middle age, and it may be remarked as a very curious circumstance, that while in the males all the attacks I have seen were once only, they have been very frequently repeated in the females. These women indeed, are so predisposed to the disease, that they scarcely ever escape it, in some degree, when subjected to its exciting causes.

Like its allied affections, an exposure to cold, and especially to a sour austere atmosphere is the usual and, perhaps, only cause of the genuine disease, and to which we have reason to believe, an increased susceptibility is created by repetitions of tonsilitis. This fact is noticed in the reports of many of the cases. But other causes have been assigned, of, at least, a variety of the disease, as whatever, in short, directly or indirectly inflames the larynx. The inhalation of the vapour of boiling water is alleged to have induced it in those who have suffered from the bursting of the boilers of steamboats, and in the instance of five children on one occasion, and on another of one, who through mistake, applied their mouths to the spout of a tea-kettle of hot water. The inflammation here, we are told, was exceedingly intense in the upper portion of the wind-pipe; attended by the prominent symptoms of Laryngitis.§ Examples too, are to be met with, of its being traced to the extension of phlogosis of the neighbouring parts, from Tonsilitis, Erysipelas, Rubeola, Scarlatina, and from Mercury.

An account is moreover given, of its having prevailed to a wide extent, spreading simultaneously over a large district of country. But I am satisfied from information on which I can rely, that the Epidemic was a species of Scarlatina Anginosa, or more in the shape of Croup, as previously shewn. Bearing a closer analogy to real Laryngitis than any of these instances, is the affection which I have noticed, of Laryngitis suddenly induced by a reflection as it would seem, of an irritation from the stomach.

Laryngitis may be distinguished from Croup by the partially, or as it may sometimes happen, diffusively inflamed Fauces, by tenderness of the Larynx, by difficulty of deglutition, by the absence

* Percival's case; vide Med. Chirurg. Transactions, vol. 4, p. 298.

† Porter, p. 97.

‡ Dr. Macnamara Hayes, and Dr. David Pitcairn.

§ These cases are reported by Dr. Marshall Hall in the 12th vol. Med. Chirurg. Trans. London, and by Dr. Burgess, in 3d vol. Dublin Hosp. Reports.

nearly of cough and peculiar raucal intonation, and lastly perhaps, by the periods of life at which they occur, the one being mostly incident to advanced age and the other to children.

Considered pathologically there is a further distinction. It will presently be seen, though in each case the Larynx is principally affected, that in Croup the mucous coat is inflamed, with an extravasation of coagulable lymph on its surface, giving to it an adventitious covering:—while in Laryngitis, with comparatively slight phlogosis of the same membrane, the sub-cellular tissue, exempt in the other affection, is most deeply implicated, ending in the effusion of fluids, productive of an edematous, or some other tumefaction of the parts. The presence, however, of the fibrous membrane in the first, and the want of it in the second, marks most strongly the individuality of the diseases.

The difficulty of discrimination is still less with regard to ordinary Cynanche Tonsillaris, another case which somewhat resembles it. Though in Laryngitis there may be redness, no great swelling exists of the tonsils or adjacent structures in the genuine disease, and we learn, that on inspection the epiglottis, from swelling, will be seen erect, and by pulling the tongue much pain is suffered, from its connection with the inflamed parts. These circumstances, or some of them, at least, equally serve to distinguish it from pharyngitis, and to which I shall add, that in the latter, while the attempt to swallow is agonising, the respiratory function remains undisturbed.

*To be Continued.*

